# Identification of anaplastic lymphoma kinase as a potential therapeutic target in Basal Cell Carcinoma

**DOI:** 10.18632/oncotarget.1357

**Published:** 2013-10-02

**Authors:** Hanna Ning, Hiroshi Mitsui, Claire Q.F. Wang, Mayte Suárez-Fariñas, Juana Gonzalez, Kejal R. Shah, Jie Chen, Israel Coats, Diane Felsen, John A. Carucci, James G. Krueger

**Affiliations:** ^1^ Laboratory for Investigative Dermatology, The Rockefeller University, New York, NY; ^2^ Center for Clinical and Translational Science, The Rockefeller University, New York, New York, USA; ^3^ Texas Dermatology Associates, Baylor University Medical Center, Dallas, TX USA; ^4^ Institute for Pediatric Urology, Department of Urology, Weill Cornell Medical College, New York, NY; ^5^ Ronald O. Perelman Department of Dermatology, New York University Langone Medical Center, New York, NY

**Keywords:** Basal cell carcinoma, oncogenic kinases, cancer, therapy

## Abstract

The pathogenesis of BCC is associated with sonic hedgehog (SHH) signaling. Vismodegib, a smoothened inhibitor that targets this pathway, is now in clinical use for advanced BCC patients, but its efficacy is limited. Therefore, new therapeutic options for this cancer are required. We studied gene expression profiling of BCC tumour tissues coupled with laser capture microdissection to identify tumour specific receptor tyrosine kinase expression that can be targeted by small molecule inhibitors. We found a >250 fold increase (FDR<10^−4^) of the oncogene, anaplastic lymphoma kinase (ALK) as well as its ligands, pleiotrophin and midkine in BCC compared to microdissected normal epidermis. qRT-PCR confirmed increased expression of ALK (p<0.05). Stronger expression of phosphorylated ALK in BCC tumour nests than normal skin was observed by immunohistochemistry. Crizotinib, an FDA-approved ALK inhibitor, reduced keratinocyte proliferation in culture, whereas a c-Met inhibitor did not. Crizotinib significantly reduced the expression of GLI1 and CCND2 (members of SHH-pathway) mRNA by approximately 60% and 20%, respectively (p<0.01). Our data suggest that ALK may increase GLI1 expression in parallel with the conventional SHH-pathway and promote keratinocyte proliferation. Hence, an ALK inhibitor alone or in combination with targeting SHH-pathway molecules may be a potential treatment for BCC patients.

## INTRODUCTION

Basal cell carcinoma (BCC) is the most common human cancer with an estimate of well over one million new cases diagnosed in the United States every year [1]. There are two main types of BCC: the less-aggressive (nodular and superficial) and the aggressive (infiltrative/morphea-like types). The morbidity from local invasion and destruction of the surrounding tissue can cause severe functional deficit and cosmetic disfigurement. However, it rarely metastasizes or causes death [2].

The sonic hedgehog (SHH) pathway is associated with BCC development [3]. This pathway is highly conserved across species, and it is responsible for controlling embryonic development and adult tissue homeostasis [4]. SHH binds to protein patched homolog 1 (PTCH1) and prevents PTCH1 from inhibiting smoothened (SMO). PTCH1 inhibits SMO from translocating to the cell membrane, thereby enhancing degradation of SMO. In the absence of PTCH1, the GLI-family proteins, key transcription factors downstream of SMO, thereby turn on SHH-responsive genes, including many prosurvival and other transcription factors that are essential for tumour growth and survival [5].

Mutations in PTCH1 can cause the development of nevoid basal cell carcinoma syndrome (NBCCS), also known as Gorlin syndrome [6]. NBCCS is a predisposition to large numbers of BCCs. NBCCS patients have an inherited mutations at least one allele of the *PTCH1* gene [6]. In sporadic BCC patients, it is also estimated that loss of function mutations in *PTCH1* occur in 30-40%, while gain of function mutations in *SMO* are found in approximately 10% [7, 8]. Both mutations result in constitutive activation of SMO.

Treatment for BCC is largely achieved by surgical excision or destruction, but there are select cases of locally aggressive BCC where surgery may be complicated by severe functional compromise. Other therapeutic options include vismodegib, a recently FDA-approved SMO inhibitor for treating advanced BCC patients, or immune activation with imiquimod. These options, however, are not effective for all BCC patients. Imiquimod can only be used in superficial BCC [9]. It is also discouraging that objective responses of vismodegib were only seen in 30% of patients with metastatic BCC [10] and 43% [10] or 58% [11] of patients with locally advanced BCC. Therefore, further research in molecular mechanisms of BCC development are needed, in order to develop better therapies.

Anaplastic lymphoma kinase (ALK) is a transmembrane receptor tyrosine kinase of the insulin receptor superfamily [12]. It plays an important role in brain and neuronal development during embryogenesis. The expression of ALK is diminished in the adult; however, it is still found in specific tissues of neuronal origin. ALK is activated by its ligands, midkine (MDK) and pleiotrophin (PTN), both of which serve as mitogenic and angiogenic factors in cancer [13, 14]. ALK was initially identified as an oncogenic driver in anaplastic large cell lymphoma [15, 16]. Chromosomal translocations, resulting in fusion oncogene of ALK have also been described in multiple cancers such as non-small cell lung cancer, inflammatory myofibroblastic tumours, and others [17-20]. Furthermore, a number of gain of function point mutations in ALK have been identified in neuroblastoma [21], pointing to the important role of ALK in driving tumour development. An ALK inhibitor, crizotinib, has been recently FDA approved as a therapy for late stage non-small cell lung cancer with little side effects [22, 23]. This makes ALK an intriguing target as a therapy for many other cancers.

In this study, laser capture microdissection (LCM) was performed in combination with cDNA microarray analysis in order to discover molecular pathways that distinguish BCC from normal epidermal keratinocytes. We found that ALK was up-regulated by >250 fold in BCC nodules and cognate activation of PTN and MDK ligands also occurred. ALK was phosphorylated in BCC tumour nests. Crizotinib reduced keratinocyte proliferation in culture in part by suppressing the expression of SHH signaling genes GLI1 and CCND2. Our data suggest that ALK activates GLI1 in parallel with the conventional SHH-pathway. Furthermore, ALK inhibitor alone or in combination with targeting the SHH-pathway molecules may be applicable for treating BCC patients.

## RESULTS

### Laser capture microdissection confirms previously identified genes using bulk tissue extracts from BCC tissue

**Figure 1:**
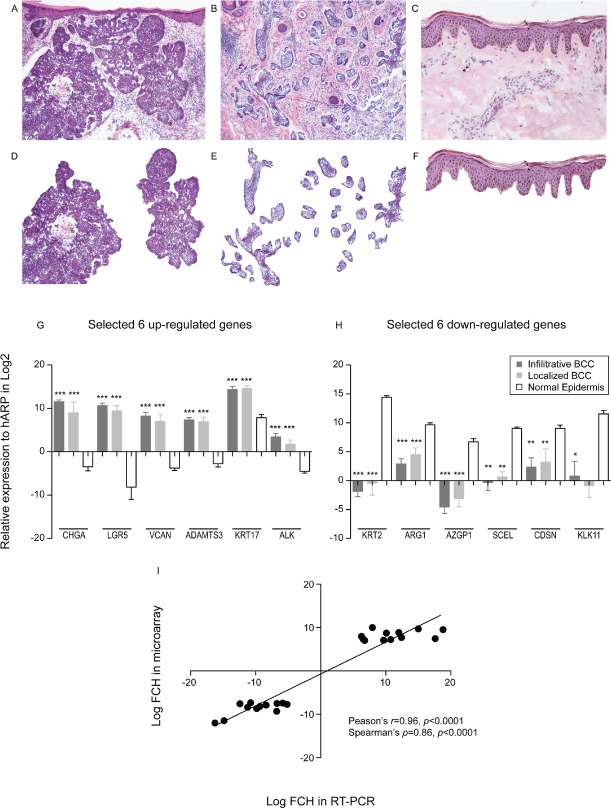
Laser capture microdissection identifies genes specific to BCC tumour nests

Laser capture microdissection was performed on both localized and infiltrative BCC (Figure 1A-F), followed by RNA extraction, target amplification and labeling, and hybridization onto Affymetrix HGU133A2.0 chips. In humans, BCC arises from the interfollicular epidermis; hence gene expression profiles of both BCC types were compared to those of microdissected epidermis from healthy volunteers. Table 1 shows selected up- and down-regulated genes among differentially expressed genes (false discovery rate [FDR]<0.05, fold change [FCH]>3.0). Many up-regulated genes in this short list confirm the results from previous microarray studies of BCC. Many keratinocyte differentiation marker genes (KRT2, FLG, LOR, LCE2B, and CDSN) were found to be down-regulated, but they were not detected in previous gene expression studies [24-26]. This may be explained by contamination of the normal epidermis within bulk tissue, thus showing the specificity of our LCM method to detect cancer cell specific gene expression changes. Six up- and down-regulated genes (genes with an asterisk in Table 1) were further tested for their mRNA expression changes by quantitative RT-PCR (qRT-PCR). All genes were confirmed to be differentially expressed (*p*< 0.05, Figure 1G-H), except for KLK11 expression in localized BCC, which failed to reach statistical significance. These findings showed a high correlation between FCH estimated by both methods (Pearson's r=0.96 and Spearman's ρ=0.86, *p*<0.0001 for both, Figure 1I). These data support the consistency of our LCM derived gene sets with previous studies using bulk tissue extracts, and the accuracy of our gene sets.

(A-F) Representative images of tissues used in this study are shown. Upper panels demonstrate the images for (A) nodular BCC, (B) infiltrative BCC, and (C) normal skin before LCM. Lower panels represent the images for (D) nodular BCC (n=6), (E) infiltrative BCC (n=5), and (F) normal skin (n=10) after LCM. (G) The expression of selected up-regulated genes from the gene lists was confirmed by quantitative RT-PCR (qRT-PCR). *** indicates *p*<0.0001 vs. normal epidermis (one-way ANOVA). (H) The expression of selected down-regulated genes from the gene lists was confirmed by quantitative RT-PCR (qRT-PCR). * indicates *p*<0.05, ** indicates *p*<0.01, and *** indicates *p*<0.0001 vs. normal epidermis (one-way ANOVA). (I) Pearson's correlation coefficient (*r*) and Spearman's rank correlation (*ρ*) were obtained for mean log FCH from qRT-PCR data (x-axis) and that from microarray data (y-axis) using twelve genes.

**Table 1:** Selected up- and down-regulated genes in laser captured BCC vs. laser captured normal epidermis

A. Selected up-regulated genes				
Gene symbol	Fold change		Description	Reference
	Localized BCC	Infiltrative BCC		
CHGA*	206.08	823.37	chromogranin A	O'Driscoll et al. 2006 [24]
LGR5*	175.01	727.07	leucine-rich repeat-containing G protein-coupled receptor 5	O'Driscoll et al. 2006 [24] Tanese et al. 2008 [25]
VCAN*	149.71	461.04	versican	O'Driscoll et al. 2006 [24]
ADAMTS3*	136.08	420.00	ADAM metallopeptidase with thrombospondin type 1 motif, 3	O'Driscoll et al. 2006 [24] Tanese et al. 2008 [25]
KRT17*	130.24	162.35	keratin 17	Yu et al. 2008 [26]
SOX11	7.06	10.15	SRY (sex determining region Y)-box 11	Tanese et al. 2008 [25]
ALK*	249.46	1015.62	anaplastic lymphoma receptor tyrosine kinase	O'Driscoll et al. 2006 [24]
PTN	46.91	35.30	pleiotrophin	N.R.
MDK	19.91	20.39	midkine	N.R.
B. Selected down-regulated genes				
Gene symbol	Fold change		Description	Reference
	Localized BCC	Infiltrative BCC		
KRT2*	2880.29	4104.53	keratin 2	N.R.
FLG	989.12	2247.34	filaggrin	N.R.
LOR	887.74	1354.93	loricrin	N.R.
SCEL*	210.40	633.85	sciellin	N.R.
ARG1*	412.71	337.79	arginase, liver	N.R.
AZGP1*	237.04	297.35	alpha-2-glycoprotein 1, zinc-binding	O'Driscoll et al. 2006 [24]
LCE2B	230.72	219.18	late cornified envelope 2B	N.R.
KLK11*	172.69	181.27	kallikrein-related peptidase 11	N.R.
HAL	197.68	174.25	histidine ammonia-lyase	N.R.
CDSN*	191.61	162.69	corneodesmosin	N.R.

A false discovery rate <10^−4^ is applied to all genes listed.

Genes with an asterisk were further confirmed by qRT-PCR method and the results were found in Figure 1G and 1H.

N.R.: Genes with no report in the previous BCC cDNA microarray studies.

### ALK is overexpressed and phosphorylated in BCC tissue

**Figure 2:**
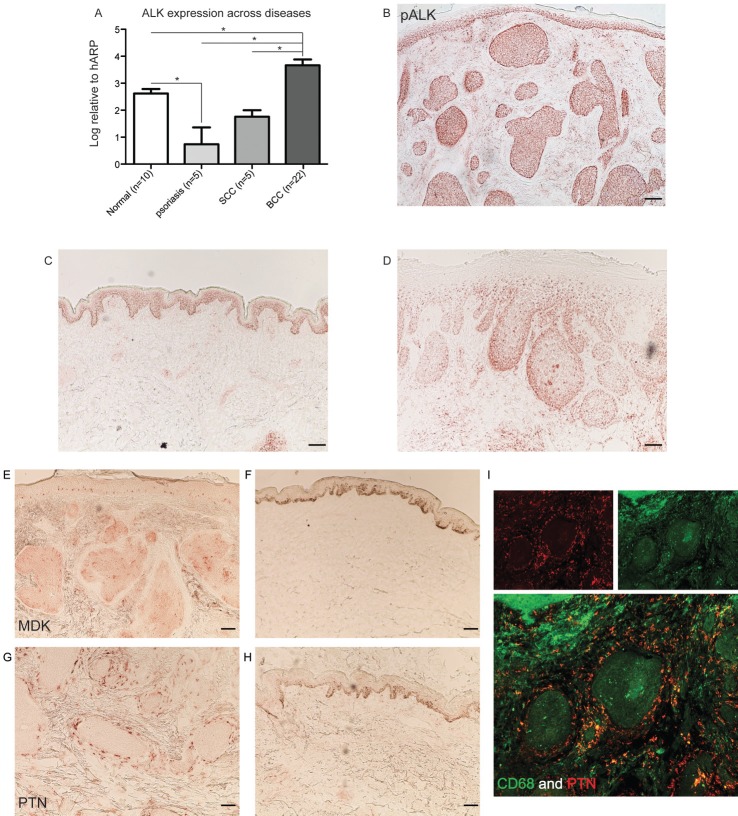
ALK and its ligands PTN and MDK are expressed in BCC tissue

ALK was found to be among the top 3 up-regulated genes for both types of BCCs with an approximately 250-FCH in localized BCC, and an approximately 1000-FCH in infiltrative BCC compared to microdissected normal human epidermis (FDR<10^−4^, Table 1A). Overexpression of ALK in microdissected BCC tissue was confirmed by qRT-PCR (Figure 1G). In order to obtain a broad view of the expression of ALK in skin diseases, qRT-PCR using total RNA extracted from bulk tissues was carried out. ALK expression was compared between different hyperproliferative skin diseases, such as psoriasis vulgaris and squamous cell carcinoma (SCC). There was a significantly higher expression of ALK in BCC compared to normal skin, psoriasis, and SCC (*p*<0.05, Figure 2A). The phosphorylation status of ALK was evaluated in BCC, as well as in SCC and normal skin. Positive cytoplasmic staining of phosphorylated ALK (pALK) in all tumour nests in BCC was found (Figure 2B). It was noted that it also stained the basal layer of keratinocytes in normal skin (Figure 2C) and peripheral regions of the SCC tumour nests (Figure 2D).

(A) mRNA expression of ALK was examined in total RNA extracted from bulk tissues across various skin conditions. SCC = squamous cell carcinoma. Error bars represent S.E.M. * indicates *p*<0.05 (one-way ANOVA). (B-D) ALK expression was examined at the protein level using a phosphorylated ALK antibody in (B) BCC, (C) normal skin, and (D) SCC. (E-F) MDK expression was examined at the protein level using a monoclonal antibody directed to MDK in (E) BCC and (F) normal skin. (G-H) PTN expression was examined at a protein level using a monoclonal antibody directed to PTN in (G) BCC and (H) normal skin. Scale bar = 100μm. (i) Double IF showing co-expression of PTN (red) and CD68 (green). Merged cells are in yellow.

### ALK ligands, MDK and PTN, are up-regulated in BCC

MDK and PTN, the two known ligands that bind to ALK, were significantly up-regulated in BCC on our gene list (FCH>20 and FCH>35, respectively, Table 1A). MDK and PTN were stained in BCC tissues by immunohistochemistry (IHC). MDK was found in cells within the tumour nests (Figure 2E), but there was no staining in normal skin (Figure 2F). PTN was present in cells at the periphery of the tumour nests (Figure 2G-H), confirming the presence of both ligands within the BCC microenvironment at the protein level. Cells responsible for the expression of PTN were then examined by double immunofluorescence (IF), showing colocalized CD68 and PTN staining; this demonstrates that CD68 positive myeloid lineage cells are producing PTN in BCC (Figure 2I).

### ALK expression is associated with active tumour growth *in vivo*

**Figure 3:**
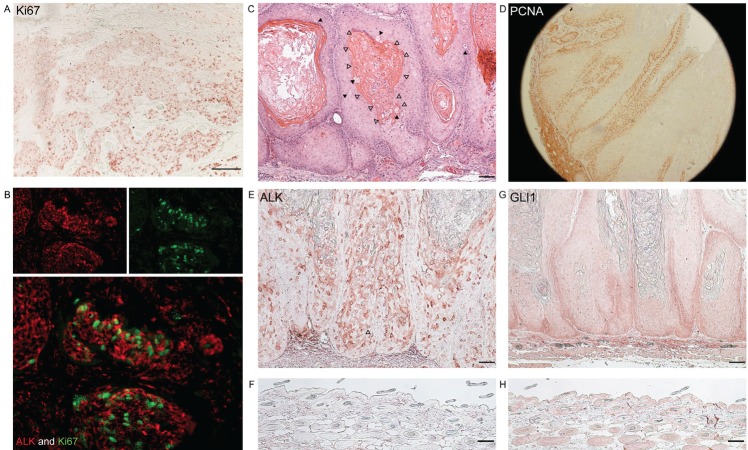
ALK and GLI1 are expressed in highly proliferating keratinocytic tumours in human and mice

The IHC results suggest that ALK might be phosphorylated in proliferating keratinocytes. In addition, the prominent expression of Ki67, a proliferation marker, in human BCC tumour nests demonstrates the highly proliferative nature of this cancer (Figure 3A). Double IF staining using antibodies for pALK and Ki67 on human BCC tissues was carried out. There was positive nuclear staining of Ki67 and cytoplasmic staining of ALK within the same tumour nests (Figure 3B). This suggests a function of ALK in tumour proliferation in human BCC.

(A) Expression of Ki67, a marker of cell proliferation, was examined at the protein level in human BCC. Scale bar = 100μm (B) Double IF showing co-expression of phosphorylated ALK (red) and Ki67 (green) in human BCC tumour nests. (C) A representative image of H&E staining of a skin tumour established in NPM-ALK transgenic mice. These skin tumour show some features of trichilemmal differentiation; absence of granular layer (white arrow heads), existence of clear cells (solid arrow heads), and architectural similarities of a tumour compartment to hair shaft. (D) PCNA, a marker of cell proliferation, was examined in the mouse skin tumour. (E-F) ALK expression was examined at a protein level in the (E) mouse skin tumour and (F) normal skin. (G-H) GLI1 expression was examined at a protein level in the (G) mouse skin tumour and (H) normal skin.

Giuriato et al. [27] have developed mice transduced with NPM-ALK or TPM3-ALK fusion genes. These mice develop skin tumours as well as lymphoma. The skin tumours show follicular differentiation as evidenced by the lack of granular layer, the existence of clear cells, and architectural similarities of tumour compartments to the hair shaft (Figure 3C). These mouse tumours are highly proliferative as evidenced by PCNA staining (Figure 3D). As expected, ALK was positive in these tumours (Figure 3E-F). GLI1 expression was also examined as the SHH-pathway is known to be involved in hair follicle morphogenesis. As shown in Figure 3G, GLI1 expression at the peripheral area of the tumour nests was increased compared to normal epidermis (Figure 3H). Overall, these results associate the expression of ALK and GLI1 with keratinocyte proliferation.

### ALK inhibitor inhibits normal keratinocyte proliferation *in vitro*

**Figure 4:**
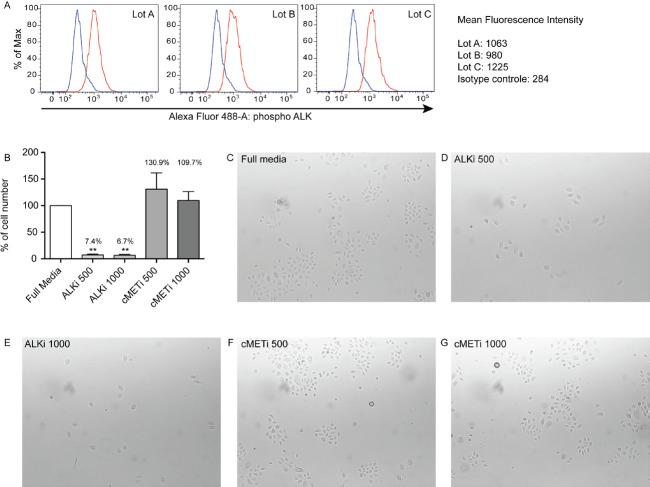
Crizotinib inhibits the growth of normal human epidermal keratinocytes

Crizotinib (PF-2341066, an ALK-inhibitor) is an FDA-approved drug for treating non-small cell lung cancer patients with a mutation in ALK. Since there are no commercially available BCC cell lines to date, the potential to inhibit proliferation of keratinocytes with crizotinib was evaluated using cultured normal human epidermal keratinocytes (NHEKs). The phosphorylation status of ALK in cultured keratinocytes was first evaluated by using flow cytometry analysis. ALK was consistently phosphorylated in all three NHEKs tested (Figure 4A). The mean of the median fluorescence intensity (MFI) was 1089, which was higher compared to the isotype control antibody with a mean MFI of 284. Cells were then cultured with or without crizotinib or a c-Met inhibitor (PF-04217403) in media with full supplements. The c-Met inhibitor at different doses was also included, as it is known that crizotinib can also inhibit c-Met activity. The number of keratinocytes in culture treated with crizotinib for 5 days was significantly lower than control (full media). A greater than 90% inhibition was observed after treatment with crizotinib at both doses (Figure 4B, *p*<0.01). The c-Met inhibitor alone did not affect keratinocyte growth (Figure 4B and 4C-G). This further supports the role of ALK in regulating proliferation of keratinocytes.

(A) Phosphorylation of ALK in normal keratinocytes is shown by flow cytometry. Cells from different three donors were cultured in full media for 5 days and the expression of phosphorylated ALK was evaluated. The histograms show an increased signal of phosphorylated ALK (red) compared to the isotype control (blue). Median fluorescence intensity for phosphorylated ALK and isotype control are shown on the right. (B) Cells were harvested after 5 days of culture with or without indicated inhibitors and their numbers were counted. The bar graph shows the ratio of the number of cells to control (full media) from the average of independent three experiments with three different donors. There were significantly fewer cells following ALK-inhibition (ALK inh) compared to cells in media with full supplements (Full media) and cells treated with c-Met inhibitor (cMET inh). ** indicates *p*<0.01 vs. full media (repeated measures ANOVA). The number on each graph shows the percentage of cell numbers compared to control (PBS). (C-G) The cells were photographed everyday. Representative images at 4 days of culture in (C) full media, (D) ALK inhibitor at 500ng/ml, (E) ALK inhibitor at 1000ng/ml, (F) c-Met inhibitor at 500ng/ml, and (G) c-Met inhibitor at 1000ng/ml are shown.

### ALK inhibitor represses the SHH-pathway in cultured keratinocytes

**Figure 5:**
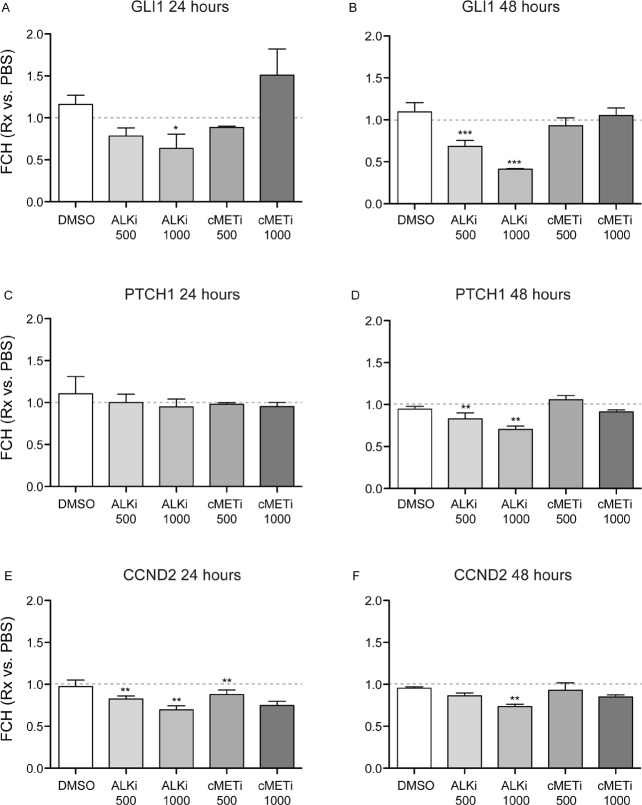
Crizotinib down-regulates the expression of the SHH-pathway genes in normal human epidermal keratinocytes

It has been reported that ALK induces GLI1 mRNA expression via PI3/Akt in a lymphoma cell line [28]. To further elucidate possible underlying mechanisms of the growth inhibition of cultured keratinocytes by crizotinib (an ALK inhibitor), mRNA expression of the SHH-pathway genes was evaluated after 24 and 48 hour treatment with indicated inhibitors and PBS as a control. The expression of each gene for each condition was normalized to that of control (PBS alone). The expression of GLI1 was significantly down-regulated in the cells after 24 hour treatment with 1000ng/ml of crizotinib (*p*<0.05, Figure 5A) and it was further suppressed after 48 hours with 500 ng/ml and 1000ng/ml of crizotinib (*p*<0.0001, Figure 5B). This suppression after 48 hour treatment with 1000ng/ml of crizotinib was obvious with an approximately 60% reduction compared to PBS. The expression of PTCH1 did not change after 24 hour culture with any treatment tested, whereas it was reduced after 48 hour culture with 500ng/ml and 1000ng/ml of crizotinib (*p*<0.01, Figure 5C-D). The expression of CCND2 was significantly down-regulated by 1000ng/ml of crizotinib after 48 hours (*p*<0.01, Figure 5F). This was also observed even in the cells after 24 hour culture with 500ng/ml and 1000ng/ml of crizotinib (*p*<0.01, Figure 5E). There was a dose dependent manner of the effect of the crizotinib to reduce each gene expression.

Cells were cultured for 24 hours with or without indicated inhibitors or dimethyl sulfoxide (DMSO). Cells were then lysed with lysis buffer and total RNA was extracted. (A-B) mRNA expression of a key transcription factor GLI1 was evaluated in cells after (A) 24 hour and (B) 48 hour culture. (C-D) mRNA expression of PTCH1 was evaluated in cells after (C) 24 hour and (D) 48 hour culture. (E-F) mRNA expression of CCND2, a GLI1 target gene, was evaluated in cells after (E) 24 hour and (F) 48 hour culture. The expression value for each gene was first normalized to hARP, and then fold change relative to control (PBS) was estimated. An error bar indicates S.E.M. * indicates *p*<0.05, ** indicates *p*<0.01, *** indicates *p*<0.0001 in the comparison of each treatment vs. control (PBS) by using repeated measures ANOVA and adjusting p values using Dunnet's method. A dotted grey line depicts the level of equal expression to control.

### The SHH-pathway genes are up-regulated in BCC tissue

**Figure 6:**
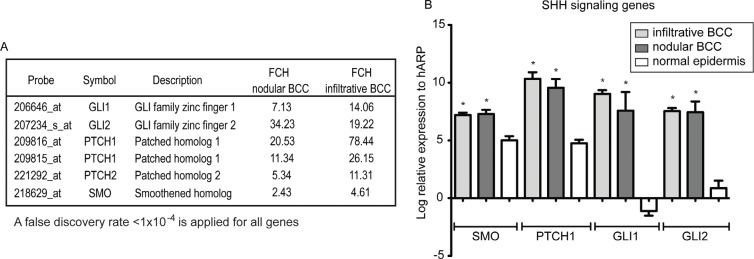
The SHH-signaling genes were up-regulated in BCC tissue

Finally, the expression of the SHH-pathway, known to be associated with BCC development, was evaluated in our nodular and infiltrative BCC transcriptomes. PTCH1, PTCH2, SMO, and key transcription factors of this pathway GLI1 and GLI2 were all up-regulated (Figure 6A). Up-regulation of four of these genes was further confirmed by qRT-PCR using the same LCM-derived RNA that we used for microarray analysis. SMO, PTCH1, GLI1, and GLI2 were all elevated in BCC compared to LCM normal epidermis (Figure 6B).

(A) The fold changes (FCH) for the SHH-pathways genes detected in our gene lists are shown. All genes satisfy false discovery rate <10^−4^. (B) mRNA expression of the selected SHH-pathway genes (SMO, PTCH1, GLI1, and GLI2) was examined in microdissected BCC tumour nests (Infiltrative and Nodular, n=5 for each group) and normal epidermis (Normal, n=5). Error bars represent S.E.M. and * indicates *p*<0.05 vs. Normal (one-way ANOVA).

## DISCUSSION

There is an increasing trend to treat specific cancers with cell-specific or pathway-specific antagonists [29-32]. In BCC, vismodegib has been recently approved by FDA for metastatic and locally advanced disease. This drug blocks the signaling from SMO to GLI1, resulting in the down-regulation of the SHH-pathway. It is well documented that the constitutive activation of the SHH-pathway, mainly due to loss of function mutations in PTCH1 or gain of function mutations in SMO, is present in the majority of NBCCS and sporadic BCC cases. However, results from first clinical trial of vismodegib were somewhat discouraging with a 30% response rate for metastatic and a 43% response rate for locally advanced BCC [10], followed by a report with a 58% response rate for locally advanced BCC [11]. In addition, drug resistance has been recently reported in 6 out of 20 locally advanced BCC patients during vismodegib treatment [33]. These data suggest that there may be alternative mechanisms that activate GLI1 in BCC. Another concern regarding the use of vismodegib is the fact that about 50% patients discontinued the drug due to its severe adverse effects [10, 34]. Thus, finding new therapeutic options for this cancer is essential.

**Figure 7:**
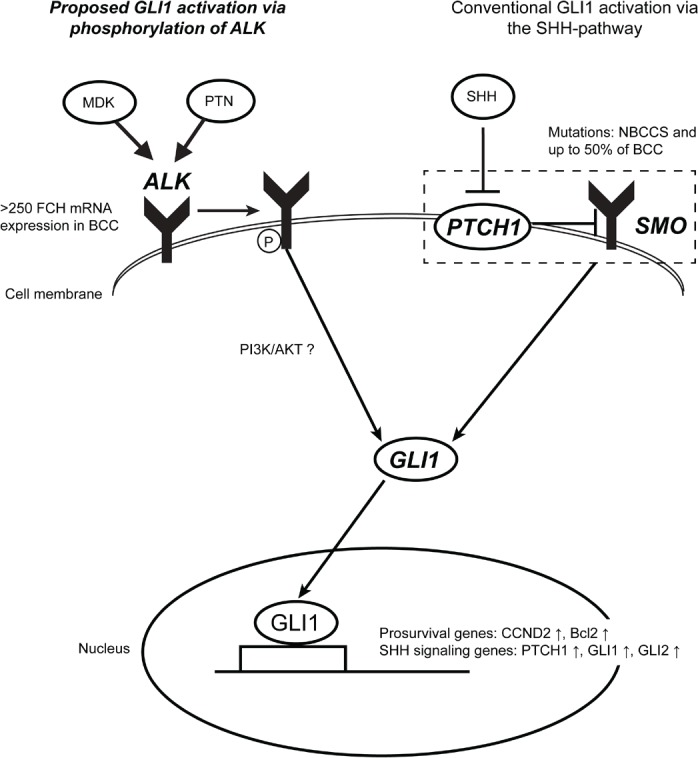
ALK may activate GLI1 in a coordinate fashion with the conventional SHH-pathway in BCC

In this study, we identified a new function of ALK in keratinocyte proliferation, as well as in activation of GLI1 signaling. The activation of GLI1 transcription factor by ALK was reported in a lymphoma cell line and was ascribed to signaling through PI3/Akt [28]. The function of ALK in keratinocytes has not been determined to date. This coordinate regulation of GLI1 expression by the conventional SHH-pathway and ALK is particularly interesting when put into the context of BCC biology. In fact, we found the overexpression of ALK mRNA in all 22 BCC cases studied. In a mouse model of BCC, it was suggested that the degree of expression of GLI1 or GLI2 has a critical impact on the development of BCC tumours [35]. In this regard, our data suggest that ALK may have a function in enhancing GLI1 expression in BCC in parallel to signaling through SMO activation. A proposed role of ALK in BCC is summarized in Figure 7.

This scheme demonstrates our model of activation of GLI1 in BCC. It is known that nevoid basal carcinoma syndrome (NBCCS) patients and up to 50% of sporadic BCC patients harbor either loss of function mutations in PTCH1 or gain of function mutations in SMO, resulting in constitutive activation of the SHH-pathway. Our data presented here showed that all (n=22) cases of BCC overexpressed ALK at a genomic level with a FCH>250. Known ALK ligands MDK and PTN exist in the BCC microenvironment at a protein level, thus ligands-receptor binding may occur. Indeed, phosphorylation of ALK in BCC tumour nests was confirmed by IHC with specific antibody against phosphorylated ALK. The effect of ALK in the regulation of GLI1 mRNA expression as well as its downstream molecule CCND2 was confirmed in normal human epidermal keratinocytes in culture with crizotinib, an FDA approved ALK inhibitor. Overall, these data suggest that GLI1 expression in BCC may be regulated by signaling through phospshorylation of ALK in parallel with the conventional SHH-pathway. This may in part explain the relatively limited response rate of vismodegib, an FDA approved SMO inhibitor, against BCC. Targeting ALK with ALK inhibitor alone or in combination with other therapies such as vismodegib may provide new therapeutic options for treating BCC patients.

The feasibility of the use of ALK inhibitor in treating BCC is also supported by mouse models. There are mice transduced with NPM-ALK or TPM3-ALK fusion genes [27]. These mutations are known to lead to constitutive activation of ALK and are found in anaplastic lymphoma patients in humans [15, 16]. Thus, these mice were established as models of anaplastic lymphoma. Unexpectedly, these mice established keratinocytic tumours on their skin as well. While analyzing these skin lesions by H&E, we noticed the trichilemmal keratinization and strong expression of ALK and GLI1 within the tumours. These features in part overlap with BCC, which is often considered as a tumour with features of hair follicle differentiation [36]. Most importantly, these tumours were completely diminished when these mice were treated with crizotinib [27]. Taken together, our observations in both human BCC and mice suggest that ALK plays an important role in BCC growth, and therefore holds great potential as a therapeutic target for BCC.

The limitation of this study is a lack of direct evidence of the effect of ALK inhibitor on human BCC cells. However, it should be noted that there are no well established and fully characterized human BCC cell lines. Mouse models of BCC do not properly recapitulate human BCC. For example, some mice BCC model harbor mutations in GLI2 [37], which is very rarely seen in humans. The structure of mouse skin features follicular differentiation of keratinocytes; hence skin tumour pathology of the ALK transgene is a reflection of this differentiation bias in mouse skin.

In summary, an oncogene ALK was highly up-regulated in BCC. Our data suggest that ALK activates GLI1 in parallel with the conventional SHH-pathway and thus these distant surface molecules may cooperate or synergize in signal transduction. Given the fact that crizotinib is an already FDA approved drug, we believe that clinical trials using crizotinib alone or in combination with other therapies such as vismodegib are indicated.

## MATERIALS AND METHODS

The detailed protocols and statistical analysis are described in the supplemental materials and methods.

### Patients and Samples

Institutional review board approval(Rockefeller University and Weill Cornell Medical University) and written informed consent were obtained before enrolling patients to participate in this study. The study was performed in adherence with the Declaration of Helsinki Principals. A total of 10 BCC samples were obtained during Mohs micrographic surgery.

### LCM

LCM was performed according to the manufacturer's protocol for the CellCut system (Molecular Machines and Industries).

### RNA extraction

Total RNA was extracted using RNeasy Micro Kit (QIAGEN).

### cDNA microarray analysis

Target amplification and labeling was performed as reported previously [38]. Affymetrix HGU133A2.0 arrays were used. The data has been deposited at the Gene Expression Omnibus repository (GSE42109).

### qRT-PCR

Standard TaqMan RT-PCR and pre-amplification RT-PCR methods (Applied Biosystems) were performed. All data were normalized to RPLP0/hARP. All primers and probes used in this study are listed in Table S1.

### IHC and IF

Frozen skin sections were prepared and standard procedures were used. Slides of NPM-ALK and TPM3-ALK transgenic mice skin tumours were kindly provided by Dr. Fabienne Meggetto (INSERM, France). ALL antibodies used in this study are listed in Table S2.

### Cell culture

NHEKs were purchased from PromoCell and cultured in the appropriate media. An ALK inhibitor (crizotinib, PF-2341066) and a c-Met inhibitor (PF-04217403) were purchased from Selleck.

### Flow cytometry

Suspensions of cultured NHEKs were stained according to standard procedures with anti-phospho ALK antibody or appropriate isotype control (Epitomics). Samples were acquired using the LSR II flow cytometer (BD Biosciences) and analyzed with Flowjo software (TreeStar Inc).

### Statistical analysis

Microarray data was analyzed using R/Bioconductor packages. The Harshlight package [39] was used to scan Affymetrix chips for spatial artifacts. Expression values were obtained using the GCRMA-algorithm. Expression values were modeled using the mixed-effect framework of Bioconductor's *limma* package. Genes with FDR<0.05 and FCH>3.0 were considered as differentially expressed genes. Repeating measures ANOVA with adjusting p values using Dunnet's method was used to evaluate the results from RT-PCR. *P*<0.05 was considered significant.

## Supplementary materials and methods


