# Supplementary material for: Identification of anaplastic lymphoma kinase as a potential therapeutic target in Basal Cell Carcinoma
Source: Oncotarget. 2013 Oct 2;4(12):2237–48. doi: 10.18632/oncotarget.1357 (PMC3926823; doi:10.18632/oncotarget.1357)
Supplement: Supplementary file 1 [file oncotarget-04-2237-s001.docx]

Supplemental materials and methods

Patients and samples

Institutional review board (the Rockefeller University and the Weill Cornell Medical University) and written informed consent were obtained before enrolling patients to participate in this study. The study was performed in adherence with the Declaration of Helsinki Principals. BCC samples were obtained during Mohs micrographic surgery. Tumour samples were immediately frozen and stored in -80^o^C.

Laser capture microdissection (LCM)

Frozen tissue sections from patients were cut at 8.0μm in a -20^o^C cryostat, and mounted on membrane mounted metal frame slides (Molecular Machines and Industries, Haslett, MI), then stored at -80^o^C. Slides were stained with Hematoxylin and Eosin (H&E) prior to LCM. LCM was performed following the manufacturer’s protocol for the CellCut system (Molecular Machines and Industries). It was completed within 2 hours after starting H&E staining, in order to prevent RNA degradation. Cells were collected in 100.0μl of RLT buffer (QIAGEN, Valencia, CA) containing 1% of β-mercaptoethanol (Fisher Scientific, Fair Lawn, NJ). The obtained samples in RLT buffer were stored in -80^o^C until RNA extraction was performed. 5 samples of each group of patients with nodular BCC and infiltrative BCC and 10 normal epidermis were included in this study. Tumour nests were laser captured microdissected from nodular and infiltrative BCC. The interfollicular epidermis was obtained from the normal skin as control (n=10). These normal epidermis samples were used for our previous study [40]. Hair follicle and hair infundibulum were avoided when laser capturing the interfollicular epidermis under microscopic visualization.

RNA extraction and quantification for microdissected cells

Total RNA was extracted using the RNeasy Micro Kit (QIAGEN) into 24μl of RNase free water. Quantity of extracted RNA was estimated using quantitative RT-PCR for RPLP0/hARP using TaqMan EZ PCR Core Reagent (Applied Biosystems, Foster City, CA). Serial dilutions of the extracted RNA ranging from 2000.0pg/μl to 1.0pg/μl were used to generate the standard curve. Samples were then amplified and quantified using 7900HT Fast Real-Time PCR System (Applied Biosystems). The thermal cycle conditions were: 2 minutes at 50^o^C; 30 minutes at 60^o^C; 5 minutes at 95^o^C; and 45 cycles of 15 seconds at 95^o^C followed by 60 seconds at 62^o^C. All assays were done in triplicates. The quality of RNA was examined using Agilent Bioanalyzer (Agilent, Santa Clara, CA).

RNA amplification and hybridization

Target amplification was performed according to the Affymetrix protocol for two-cycle cDNA synthesis with a slight modification [38]. Briefly, 1ng of total RNA extracted from microdissected tissues was subjected to amplification. SuperScript III (Invitrogen, Carlsbad, CA), instead of SuperScript II was used. The total RNA/T7-oligo (dT) mix was incubated at 65^o^C for five minutes, and then it was incubated for one hour at 50^o^C for First-Cycle, First Strand cDNA Synthesis. In the second cycle, non-labeled cRNA was incubated with Random Primers for 5 minutes at 65^o^C, and SuperScript III was added to synthesize first-strand cDNA for one hour at 50^o^C. Labeling of cRNA transcripts with biotin was performed using the GeneChip IVT Labeling Kit (Affymetrix, Santa Clara, CA). Fifteen μg of biotin-labeled RNA was fragmented and hybridized to HGU133 A2.0 arrays (Affymetrix), washed, stained, and scanned according to the manufacturer’s protocol. Affymetrix Human Genome U133A 2.0 Array comprises of over 500,000 unique oligonucleotide features covering over 18,000 transcripts and variants including 14,500 of the best characterized human genes.

Quantitative RT-PCR

Pre-amplification RT-PCR technique was used for detecting the selected six up- and six down-regulated genes as well as the SHH-pathway genes on RNA extracted from microdissected BCC and normal epidermis. 5ng of total RNA was subhected to cDNA synthesis using High Capacity cDNA Reverse Transcription Kit (Applied Biosystems). Conditions for the thermal cycler were: 10 minutes at 25^o^C, 120 minutes at 37^o^C, 5 minutes at 85^o^C and finally cooled down to 4^o^C. The resulting cDNA was subjected to 14 cycles of pre-amplification reaction using TaqMan PreAmp Master Mix (Applied Biosystems) with designed pooled assay mix. Pre-amplification mode was: 10 minutes at 95^o^C, and 14 cycles of 15 seconds at 95^o^C and 4 minutes for 60^o^C. 12.5μl of pre-amplified cDNA was used to measure each gene expression using TaqMan Gene Expression Master Mix and TaqMan Gene Expression Assay (Applied Biosystems) for each target gene. The thermal cycle conditions using 7900HT Fast Real-Time PCR System (Applied Biosystems) were: 2 minutes at 50^o^C; 5 minutes at 95^o^C; and 40 cycles of 15 seconds at 95^o^C followed by 60 seconds at 62^o^C. All assays were done in duplicates, and all data was normalized to RPLP0/hARP.

Regular TaqMan RT-PCR measured ALK expression, in normal skin (n=10), psoriasis (n=5), squamous cell carcinoma (n=5), and nodular BCC (n=22). Ten ng of total RNA was mixed with TaqMan Gene Expression Master Mix (Applied Biosystems) and ALK primer. Reaction was performed using 7900HT Fast Real-Time PCR System (Applied Biosystems) and the thermal cycler condition was following: 2 minutes at 50^o^C, 30 minutes at 60^o^C, 5 minutes at 95^o^C followed by 40 cycles of 15 seconds at 95^o^C and 1minutes at 62^o^C. All data was normalized to RPLP0/hARP. All primers and probes used in this study can be found in Table S1.

Immunohistochemistry

Immunohistochemistry was performed on frozen sections of human BCC samples (n=10) and normal skin (n=5). Slides were fixed in cold aceton for 2 minutes and washed with PBS. 10% normal blocking serum of horse or goat was used to block the slides for 20 minutes at room temperature in a humidified chamber. Samples were then incubated overnight at 4^o^C with the primary antibody diluted in 1% normal blocking serum. Biotin labeled horse anti-mouse and biotin-labeled goat anti-rabbit (Vector Laboratories, Burlingame, CA) were used to detect the primary antibodies. Slides were incubated for 30 minutes with the biotinylated secondary antibody diluted in 1% normal blocking serum. Following the incubation, slides were quenched of endogenous peroxidases with 0.3% H_2_O_2_ for 15 minutes. The staining signal was amplified using avidin-biotin complex (Vector Laboratories) for 30 minutes at room temperature and developed using chromogen 3-amino-9-ethylcarbazole. Stained samples were observed, and pictures were taken under a Nikon Eclipse 50i microscope with a Nikon DS-Fi1 camera (Nikon, Melville, NY). Antibodies used in this study can be found in Table S2.

Immunofluorescence

Human BCC sections were fixed in acetone for 3 minutes and blocked with 10% normal goat serum (Vector Laboratories) for 30 minutes. Primary antibodies pALK and Ki67 were incubated overnight at 4^o^C. Fluorochromes, goat antirabbit Alexa 568 or goat anti-mouse Alexa 488 was added to the sections for 30 minutes. Images were obtained through a Zeiss Axioplan 21 microscope with Plan Apochromat 20x0.7 numerical aperture lens and a Hamamatsu Orca ER-cooled charge-coupled device camera, accompanied by METAVUE software (Molecular Devices, Sunnyvale, CA). The same process was conducted for double IF for CD68 and PTN. Cells that co-express both markers are colored yellow showing colocalization. Antibodies used in this study can be found in Table S2.

Immunohistochemistry on mouse tumour tissues

Slides of NPM-ALK and TPM3-ALK transduced mouse skin tumors were kindly provided by Dr. Fabienne Meggetto (Institut National de la Santé et de la Recherche Médicale [INSERM], France).

For ALK and GLI1 staining, formalin fixed paraffin embedded mouse tumour slides were deparaffinized with three incubations of Citrisolve (Fisher Scientific, Fair Lawn, NJ), followed by rehydration in decreasing concentrations of ethanol. For antigen retrieval, slides were heated in Diva Decloaker, RTU (Biocare Medical, Pike Lane Concord, CA) for 30 minutes at 90^o^C.  Following the antigen unmasking, slides were quenched of endogenous peroxidases with 0.3% hydrogen peroxide for 10 minutes. 10% normal goat serum of was used to block the slides for 30 minutes at room temperature in a humidified chamber. Samples were then incubated overnight at 4^o^C with the primary antibody diluted in 1% normal goat serum. Biotin labeled goat anti-mouse and biotin-labeled goat anti-rabbit (Vector Laboratories, Burlingame, CA) were used to detect the primary antibodies.  Slides were incubated for 60 minutes with the biotinylated secondary antibody diluted in 1% normal goatg serum.  The staining signal was amplified using avidin-biotin complex (Vector Laboratories) for 45 minutes at room temperature, and developed using chromogen 3-amino-9-ethylcarbazole.

For PCNA staining, sections were deparaffinized with Hemo-De (Scientific Safety Solvent, Keller, TX) for 30 minutes, rehydrated in decreasing concentrations of ethanol, and washed three times in PBS for 10 minutes. Endogenous peroxidase was quenched for 30 minutes with 0.3% hydrogen peroxide in methanol. To retrieve antigen, slides were boiled with 10 mmol/L citrate buffer (pH 6.8) for 10 minutes. After washing in filtered water and PBS, a blocking step was included using 5% species-appropriate serum in conjunction with avidin and biotin blocking solutions (Vector Laboratories) for a total of 30 minutes. Primary antibody to PCNA (Dako, Via Real Carpinteria, CA) was then applied at room temperature for one hour. Biotinylated rabbit anti-mouse secondary antibody (Vector Laboratories) was incubated for 30 minutes. Sections were then incubated with avidin-biotin peroxidase complex (Vector Laboratories) and developed with diaminobenzedene.

All antibodies used in this study can be found in Table S2.

Normal human epidermal keratinocytes culture

Normal human epidermal keratinocytes (NHEK) from three different donors were purchased from PromoCell and suspended in keratinocyte growth medium 2 (Promocell, Germany). Cells were plated in media with all supplements and the indicated amount of ALK inhibitor (crizotinib [PF-2341066], Selleck, Houston, TX) or c-Met inhibitor (PF-04217403, Selleck) were added 24 hours after plating. A full supplement includes bovine pituitary extract, epidermal growth factor (EGF), insulin, hydrocortisone, epinephrine, transferrin, and CaCl_2_ (all supplied by Promocell).

The NHEK cells were cultured for 5-6 days at 37^o^C in a humidified atmosphere containing 5% CO_2_ in order to evaluate the efficacy of the inhibitors in inhibiting cell proliferation. The morphology of the colonies was observed under a Zeiss microscope with an attached Jenoptik camera. Photos were taken every day of the colony growth for each circumstance. After 5 days, the cultured NHEK were harvested with 0.25% trypsin, and counted manually with counting chamber (Hausser Scientific, Horsham, PA) after staining with trypan blue (Thermo Scientific, Kalamazoo, MI). The experiments were repeated three times with three different donors.

The NHEK cells in the above conditions were cultured for 24 hours or 48 hours in order to evaluate the efficacy of the inhibitors in inhibiting the SHH-pathway gene expression.

Flow cytometry:

NHEKs were plated in 6-well plates at 1000 cells/cm^2^ in media with full supplements for 5 days. The cells were detached from the plate using dissociation solution (non-enzymatic, C5914 Sigma-Aldrich, St. Louis, MO), and were incubated for 10-30 minutes at 37^o^C. When the cells were detached from the wells, PBS and 2mM EDTA were added. Cells were collected in a tube and centrifuged and washed in staining buffer (SB, PBS with 0.5% BSA and 2mM EDTA). After washing, the cells were fixed with formaldehyde (Fix buffer I, BD Bioscience, San Jose, CA) and permeabilized with 90% methanol (Perm Buffer III, BD Biosciences). The cells were resuspended in 200μl of SB. 10^5^-10^6^ cells were transferred into a 96 well plate, and centrifuged at 2000 rpm for 5 minutes. Anti-phospho ALK antibody (rabbit IgG Ab, Epitomics, Burlingame, CA, 1:100) or isotype control for rabbit IgG (Epitomics, 1:500) in 100μl were added to the cells, and incubated for 30 minutes at room temperature. Cells were washed with SB and centrifuged. Secondary antibody, goat anti-rabbit IgG DyLight488 (Epitomics, 1:100) in 100μl was added, and incubated for 30 minutes at room temperature. Finally, the cells were washed twice with SB, and resuspended in SB for acquisition and analysis with the LSR II flow cytometer (BD Biosciences) and the FACS Diva software (BD Biosciences). The data was analyzed with Flowjo software (TreeStar Inc, Ashland). This experiment was repeated 3 times with different cell donors.

Statistical analysis

cDNA microarray data from microdissected tissue was analyzed using R/Bioconductor packages ([www.rproject](http://www.rproject).org). The Harshlight package described in Suárez-Fariñas et al. [39] was used to scan Affymetrix chips for spatial artifacts. Expression values were found using gcrma-algorithm. Probe-sets with standard deviation larger than 0.2 and expression values above 3 for at least 3 samples were included in the analysis. Expression values were linearly modeled in the limma package framework. For the comparison of interest, the moderated t-test was used to assess differential expression. P-values for each comparison were adjusted for multiple hypotheses using the Benjamini-Hochberg approach. Genes with false discovery rate <0.05 and fold change >3.0 were considered as differentially expressed genes.

All analyses from the qRT-PCR data were performed using GraphPad Prism (GraphPad Software, La Jolla, USA) with repeated measures ANOVA unless otherwise stated. P values of 0.05 and less were considered significant.

Reference

40. Kennedy-Crispin M, Billick E, Mitsui H, *et al*. Human keratinocytes have a response to injury that upregulates CCL20 and other genes linking innate and adaptive immunity. *J Invest Dermatol* 2012; 132 (1): 105-113

Table S1. A list of primers and probes used in this study

| Gene symbol | GenBank accession number |  | sequence |
| --- | --- | --- | --- |
| RPLP0/hARP | NM_001002 | forward | CGCTGCTGAACATGCTCAA |
|  |  | reverse | TGTCGAACACCTGCTGGATG |
|  |  | probe | 6-FAM-TCCCCCTTCTCCTTTGGGCTGG-TAMRA |
|  | | Assay ID | |
| ADAMTS3 | NM_014243.2 | Hs00610755_m1 | |
| ALK | NM_004304.4 | Hs01058316_m1 | |
| ARG1 | NM_000045.2 | Hs00968977_m1 | |
| AZGP1 | NM_001185.3 | Hs00426651_m1 | |
| CCND2 | NM_001759.3 | Hs00277041_m1 | |
| CDSN | NM_001264.4 | Hs00169911_m1 | |
| CHGA | NM_001275.3 | Hs00900370_m1 | |
| GLI1 | NM_001160045.1 | Hs00171790_m1 | |
| GLI2 | NM_005270.4 | Hs01119974_m1 | |
| KLK11 | NM_001136032.2 | Hs01100849_m1 | |
| KRT2 | NM_000423.2 | Hs00907210_m1 | |
| KRT17 | NM_000422.2 | Hs00356958_m1 | |
| LGR5 | NM_003667.2 | Hs00969422_m1 | |
| PTCH1 | NM_000264.3 | Hs00970979_m1 | |
| SCEL | NM_001160706.1 | Hs01557105_m1 | |
| SMO | NM_005631.4 | Hs01090242_m1 | |
| VCAN | NM_001164097.1 | Hs01007938_m1 | |

All primers and probes were purchased from Applied Biosystems.

Table S2. A list of antibodies for immunohistochemistry and immunofluorescence

| Antigen | Manufacturer | Clone ^a^ | Isotype | Dilution | Amplification-detection ^c^ |
| --- | --- | --- | --- | --- | --- |
| ALK | abcam | ALKc | IgG1 | 1/25 |  |
| pALK | Epitomics | EP661Y ^b^ | IgG | 1/5000 | Alexa 564 anti-rabbit IgG |
| CD68-FITC | DakoCytomation | KP1 | IgG1 | 1/10 | Alexa 488 anti FITC |
| GLI1 | Santa Cruz | rabbit polyclonal | IgG | 1/50 |  |
| Ki-67 | Santa Cruz | MIB-1 | IgG1 | 1/50 | Alexa 488 anti-mouse IgG1 |
| MTK | Novus Biologicals | EP1143Y ^b^ | IgG | 1/250 |  |
| PCNA | DakoCytomation | PC10 | IgG2a | 1/100 |  |
| PTN | Millipore | 3B10 | IgG1k | 1/500 | Alexa 564 anti-mouse IgG1 |

a: Antibodies used in this experiment were mouse monoclonal unless stated otherwise.

b: These were monoclonal antibodies raised in rabbit.

c: All amplification/detection antibodies were purchased from Invitrogen/Molecular Probes.
